# Trends of Rural Tropospheric Ozone at the Northwest of the Iberian Peninsula

**DOI:** 10.1100/2012/603034

**Published:** 2012-04-29

**Authors:** S. Saavedra, A. Rodríguez, J. A. Souto, J. J. Casares, J. L. Bermúdez, B. Soto

**Affiliations:** ^1^Department of Chemical Engineering, University of Santiago de Compostela, 15782 Santiago de Compostela, Spain; ^2^Department of Environment, As Pontes Power Plant, Endesa Generación S.A., 15320 La Coruna, Spain

## Abstract

Tropospheric ozone levels around urban and suburban areas at Europe and North America had increased during 80's–90's, until the application of NO*_x_* reduction strategies. However, as it was expected, this ozone depletion was not proportional to the emissions reduction. On the other hand, rural ozone levels show different trends, with peaks reduction and average increments; this different evolution could be explained by either emission changes or climate variability in a region. In this work, trends of tropospheric ozone episodes at rural sites in the northwest of the Iberian Peninsula were analyzed and compared to others observed in different regions of the Atlantic European coast. Special interest was focused on the air quality sites characterization, in order to guarantee their rural character in terms of air quality. Both episodic local meteorological and air quality measurements along five years were considered, in order to study possible meteorological influences in ozone levels, different to other European Atlantic regions.

## 1. Introduction

Apart from the stratospheric ozone, around 10–15% of the natural produced atmospheric ozone is located at the troposphere [1]. In addition, global ozone data show an increment of the ozone concentration with height, as an indicative of higher stratospheric-tropospheric exchange, and a more efficient ozone production in the upper troposphere.

At lower troposphere, the analysis of ozone ground level concentration measurements at rural and suburban areas in North America and Europe shows a typical diurnal cycle in rural sites, with minimum levels at sunrise and maximum between afternoon and evening. This pattern comes from the photochemical activity along the day, which is more favorable when low thermal inversions and soft winds keep the pollutants close to the ground level during hours.

On the other hand, sites close to large NO sources produce significant nocturnal low ozone, because of its fast reaction with NO; this can be extended to urban areas with spare but significant NO nocturnal emissions, producing strong variations of the ozone levels along every day. A different situation is usually observed downwind the city emissions, because NO has time enough to produce NO_2_ and, after that, NO_2_ is photolized by solar radiation to promote ozone production. In fact, the highest ozone peaks are usually observed in suburban and rural areas around the big cities, with hourly averages that can achieve 400–800 *μ*g/m^3^; on the other hand, isolated rural sites usually achieve lower ozone levels around 40–80 *μ*g/m^3^.

Long-term trends in tropospheric ozone are not only affected by emissions but they can also be masked by meteorological conditions. However, an annual increment of 1-2% at Europe in the period 1958–1988 was reported [2], which is coincident to similar trends in Asia [3] and Pacific [2]. At Northern Hemisphere, these changes increased the ozone levels over the Northern Atlantic [4] producing persistent levels of ozone at rural and urban areas of the European Atlantic Coast: Mace Head rural site (Ireland) increased its ozone levels in 0.14 ppb in the period 1987–1997, keeping constants after 2000 [5]. Similar trends can be observed at the US Pacific Coast [6].

At Europe, peak levels of ozone at UK decreased around 30% in the period 1986–1999 [7], but annual concentration showed a small increment. Derwent et al. [8] explained the peaks decrease because of the trends of VOCs levels during 90's. Similar results were obtained by Solberg et al. [9] at Nordic countries during 90's, probably due to the reduction of the continental emissions. However, the EMEP rural sites data are not consistent with these regional trends in the period 1990–2006, due to three different factors that affect the rural ozone levels: (a) reduction of ozone peaks, (b) reduction of NO emissions from transport and other sources, with less ozone destruction, and (c) increase of the background ozone levels at Hemispheric and global scale. As an additional factor, not yet quantified, climate change can move these trends to either positive or negative [10].

On the other hand, at cities where NO*_x_* road traffic emissions decreased, winter ozone levels increased (less ozone destruction by NO), but summer peaks decreased. Therefore, ozone urban cycles at these cities are more similar to suburban and rural patterns.

At the Iberian Peninsula, tropospheric ozone is detected in all regions and, particularly, in mediterranean and other coastal areas. This is related to the industrial development, road traffic increment, and the high isolation in central, east, and south regions. However, ozone levels distribution is very heterogeneous between regions, because of their strong differences in climate patterns, emissions profiles, and, even, possible external apportioning.

From the Iberian regions, Mediterranean has been the most widely studied, because emissions and high isolation are usually high, especially during summertime. Works of Millán et al. [11–14], Sanz and Millán [15], Alonso et al. [16], Gangoiti et al. [17, 18], and Sanz et al. [19] explained the influence of both synoptic and local coastal patterns in the ozone levels at this region. Other authors supported their studies in this region with photochemical modeling, at urban [20–22] and regional scales [23–25]. Finally, several studies were focused on topographic and land use effects [23, 26–30]. Some of their results were extended to the Cantabric [16] and Atlantic Coasts [21].

In other Iberian regions close to the northwest, some studies about tropospheric ozone were published. Different studies over rural and urban coastal areas of the Portuguese Atlantic Coast show the influence of sea breezes on the transport of ozone precursors and the production of ozone at the coastal line [31, 32], including topographic effects [33]. At rural areas, the sensitivity of ozone to BVOCs levels was also considered. Other works were focused on the Lisbon urban area [21, 34].

Cantabric Coast is also a region with significant ozone levels, mainly because the transboundary transport of ozone from the East (Central Europe). In fact, most of the studies were centered in the Cantabric Eastern Coast. Long-range transport studies [16, 18] based in field experiments were completed with the application of dispersion models [35, 36], establishing the ozone transport along stable nocturnal layers from Central Europe and, even, from Central Iberia. Finally, some studies were focused on the meteorological patterns associated to ozone peaks [37, 38].

On the other hand, at the northwest of the Iberian Peninsula, just a few of local studies were done, over industrial [41, 42] and urban areas [43]. However, due to its geographical location and climate variability, rural ozone levels at this region show a complex variability (Table 1), as they are affected by coastal meteorology, complex terrain, transboundary transport (including Atlantic background ozone transport), and, even, local ozone production at summertime. In this work, a study of the rural ozone trends along 6 years were done, based on the analysis of meteorological and air quality time series from suburban and rural sites. The problem was focused on five sites located at the northwest of this region, although measurements from surrounding areas were also considered. Particularly, a specific classification of the sites was done, in order to guarantee a representativeness of their measurements.

## 2. Area under Study

**Figure 1:**
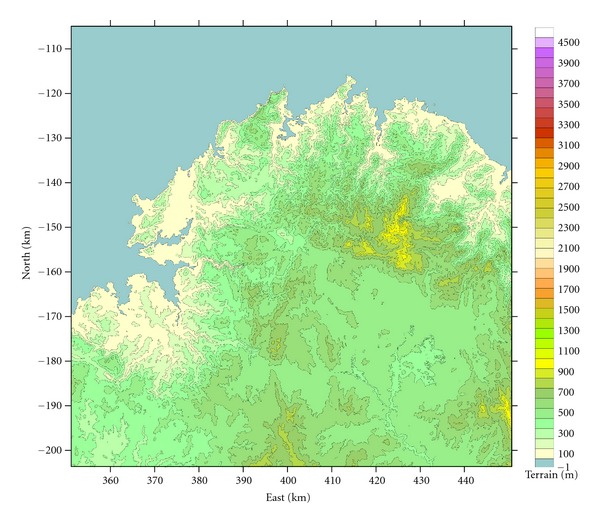
Topography of the area under study.

For the analysis of ozone trends in the extreme northwestern corner of the Iberian Peninsula, five air quality monitoring stations were selected. We analyzed hourly O_3_, NO_2_, and NO*_x_* data recorded from those sites between 2003 and 2008. This area is centered on As Pontes Valley (Figure 1); it comprises the roughly E-W-oriented lowlands around the River Eume, together with the surrounding geographic features: to the east and north mountain ranges, which reach an altitude of 1000 m; to the north, a series of hill ranges running roughly N-S from the coast, with maximum altitudes of 550–750 m; to the west, low coastal hills (<200 m) bordering the Atlantic; to the south maximum altitudes of 750–850 m; to the south east, communicating with the river Eume, the high plain of Terra Chá. Therefore, it is a complex terrain, with several granitic mountains and valleys mixed in the same environment. 

The northwestern part of Iberian Peninsula has a wide-range climate because it is a transitional zone between Oceanic and Mediterranean regimes, but the area under study is a coastal Atlantic zone with mainly Atlantic climate. The Atlantic climate is characterized by mild temperatures, with small annual temperature oscillations and abundant rainfall [44]. This region is characterized by rains distributed along the year, with an annual precipitation rate between 1000 and 1600 mm, more usual during Autumn and Spring and sporadic during summer, but not unusual, as isolated storms in the afternoon. Summers are mild, as the sea breeze refreshes the coastal areas and the altitude regulates the temperature in the inner areas. Summer days are usually sunny, with low moisture and maximum temperatures from 20 to 30°C. On the other hand, heat waves are not usual in summer, and they only spend a few days, as the proximity of the coast keeps the average temperature at 20–25°C, with higher temperatures (up to 30°C) at the inner valleys. Main winds come from the SW and NW during Winter and Autumn, with low-pressure conditions; on the other hand, high-pressure conditions typical during summertime usually correspond to NE winds. The atmosphere-topography interactions are very important in this environment, specially the mountains, with their effects in rain production, and the complex coastal topography that increases the sea breeze [45].

## 3. Classification of Air Quality Sites

**Figure 2:**
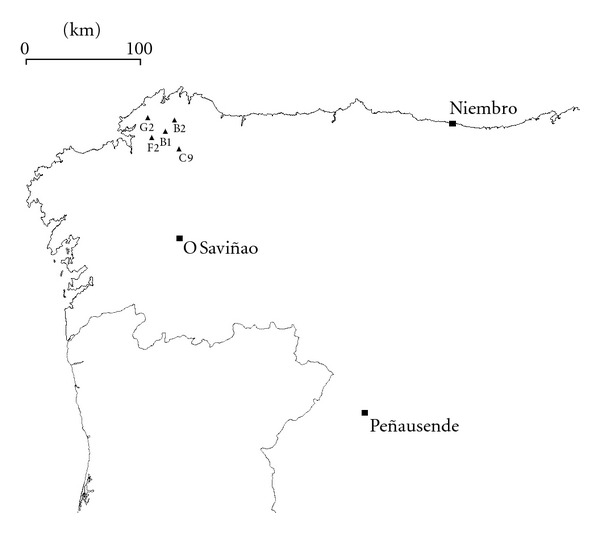
Map of the monitoring sites in NW Iberian Peninsula.

We selected seven monitoring stations (Figure 2, Table 2) as representative of the main types of stations (rural and suburban). They cover a variety of environmental conditions ranging from near-sea level to 1000 m of altitude and from sea shore to 240 km inland. 

All data analysis are based upon the hourly averaged data.  Table 3 shows the percentage of ozone data coverage. The data capture is optimal in most of the stations, higher than 95% overall, except in 2007 when the percentage of valid data covers only January–August (eight months); therefore 2007 data were not applied in all the statistics. 

The analysis of air quality time series has to take into account all the information about the location of the air quality measurements, in order to get representative values of the pollutants concentrations and to understand possible local phenomena that affect the ozone levels. 

An air quality site can be classified depending on the pollutants levels to be measured, its main goal (environmental and health protection, research), and its surrounding environment [46]. For the purposes of this work, the last criterion is the most appropriate, as measurement should be representative of a rural or, sometimes, a suburban environment where local sources influence is well characterized. However, different classifications can be considered.

- Decision 97/101/CE [47] proposes nine site types, as a combination of the land use (urban, suburban, and rural) and the main local sources (transport, industry, or none of them, as a background site).
- In addition, the topic center ETC-AQ [48] distinguished different subclasses for the background sites: urban/suburban, near-city, regional, and isolated stations.
- For ozone, Directive 92/72/CE [49] established three different sites: street, urban, and rural; and Directive 2008/50/EC [39] distinguishes background rural, rural, suburban, and urban sites.

For the five northwestern sites used to identify ozone peaks, Directive 2008/50/EC [39] was applied, because of our interest in identifying the rural stations following the most recent European methodology. First, a short description of every site location is introduced; after that, sites classification based on their ozone and precursors time series is shown. 

### 3.1. Surrounding Sites Environment

**Figure 3:**
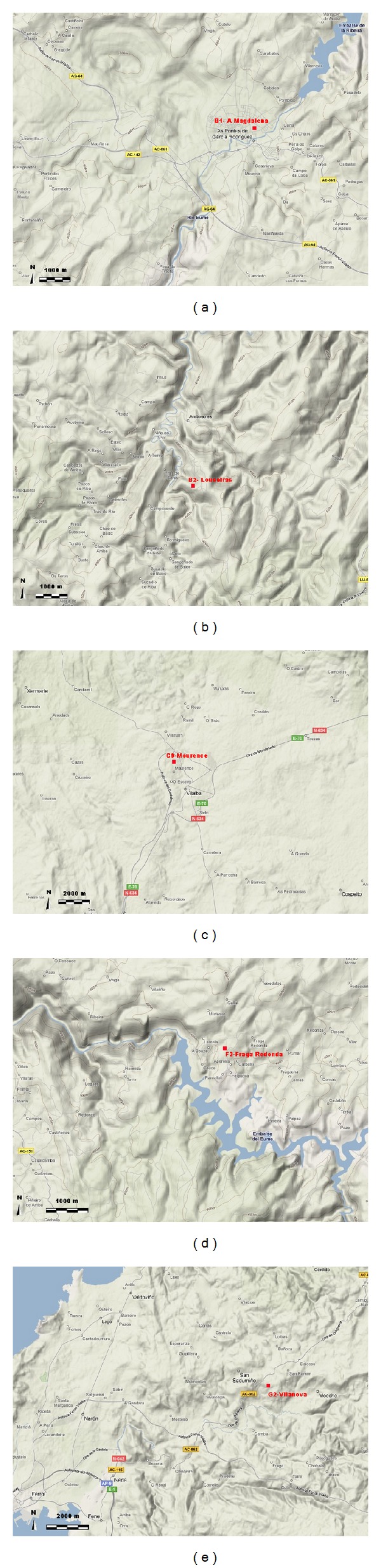
Geographic location and topography of the analyzed Galician air quality stations.

B1 site (Figure 3(a)) is located at 363 asl-m, in the small town of As Pontes, surrounded by cultived and built lands; therefore, it can be considered as a suburban site. This town is located in a valley, with an SSW drainage following the River Eume, and surrounded by small elevations (500–600 asl-m).

B2 site is located in a complex terrain area (Figure 3(b)), at 540 asl-m, in the western side of a hill close to its top (601 asl-m). A narrow valley close to the site started in the SE-NW direction, changing to SSW-NNE, creating a complex terrain environment with multiple soft hills and valleys. 

C9 site (Figure 3(c)) is located at the NW of Vilalba small town, close to it (1.5 km), so it is classified as suburban; in addition, several roads are near this site. However, the surroundings are mainly grass and cultivated land. It is located in a plateau (465 asl-m) with elevations (1000 asl-m) at 11 km to the northeast; this feature is significant, as typical northeastern dry winds in the region are softer at this site. 

G2 site (Figure 3(d)) seems to be located in a rural area, with grass and cultivated land, and wood production. However, this area is surrounded by coastal towns and industrial areas, with a significant road network; so, emissions from these sources affect this site, changing its typical rural air quality pattern. About its topography, the site is located in a valley (290 asl-m) with soft hills around it; the closest tops are located to the south at 7 km (600 asl-m) and to the east at 14 km (800 asl-m). However, it is the proximity to the coast the main factor that affect the wind on this site, with sea breezes that mix to the soft valley breezes. 

F2 site (Figure 3(e)) is located in an Atlantic forest natural park, a typical rural area, close to the Eume river dam. This zone is dominated by the complex terrain, with the site located in the east side of the dam valley (480 asl-m), that crosses from East to West and, following the river, to the coast. Winds are dominated by valley and sea breezes that usually come from the coast, 15 km far, following the river path; however, they are strongly affected by the local topography, as it will be shown. 

### 3.2. Sites Classification

In this work, the standard EU air quality sites level 1 classification [49] was applied, which considers the human activities, land use, and local emissions surrounding the site. Apart from this subjective classification based on the direct analysis of the local environment around the site, an objective analysis of air quality data can help to establish the site type [46]. For O_3_ sites, Fromage [50] used the O_3_/O*_x_* ratio, with O*_x_* = O_3_ + NO_2_, that is,

- urban sites: low O_3_/O*_x_* ratio along the year, from 0.10 (winter) to 0.50 (summer), due to the strong influence of the local NO*_x_* emissions;
- rural-regional sites: high variability in the O_3_/O*_x_* ratio, from 0.20 (winter) to 0.95 (summer), that is, O_3_ is significant in summer, but in winter the urban NO*_x_* emissions near the ratio;
- background rural sites: O_3_/O*_x_* is close to 1.00 along the year.

**Figure 4:**
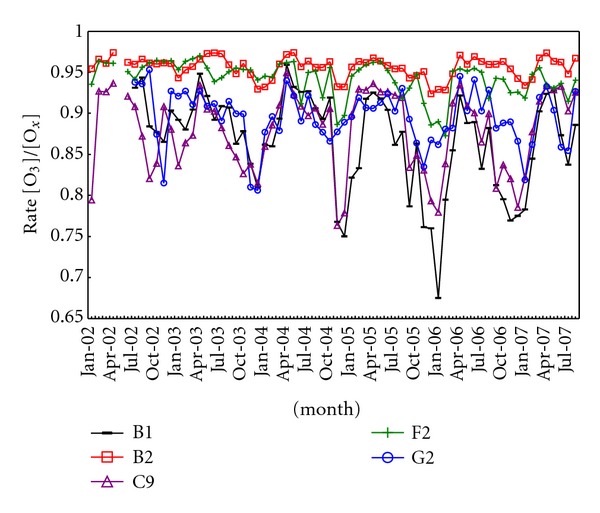
Monthly rate [O_3_]/[O*_x_*], with O*_x_* = O_3_ + NO_2_, at five monitoring stations (B1-A Magdalena, B2-Louseiras, C9-Mourence, F2-Fraga Redond, and G2-Vilanova) during the period from January 2002 to August 2007.

Figure 4 shows the results of this analysis applied to the five sites described above, for the period January 2002 to August 2007. At the same time, classification based in the sites environment [51] shows different results, as it is shown on Table 4. 

The objective classification can facilitate the accurately setting of each station to a given category, only based on the analysis of the data series of O_3_ and NO_2_, which is very useful when working with sites in unknown environments. However, the use of the subjective classification requires a thorough knowledge of the surroundings of the station, which is not always available. Moreover, the types of stations considered in the objective classification (urban, rural-regional, and rural background) are too generic to characterize a site, especially for rural stations-regional type: the criterion of high variability in the O_3_/O*_x_* ratio (0.20 to 0.95) seems too loose and bring together some sites affected by different factors (proximity to a city, a highway, or industrial sources) that should be taken into account in the analysis of ozone concentrations. This is an advantage of the subjective classification which directly considers the influence of these factors.

In the analyzed sites (Table 4), both classifications show significant discrepancies in four of the eight stations (highlights O Saviñao and G2-Vilanova stations). According to the subjective classification, O Saviñao is an EMEP site so it is a “rural background site” by definition, but objective classification assigns it to the “rural-regional” type. In this case, we have doubts about the rightness of the subjective classification applied to this EMEP station because O Saviñao often shows a marked diurnal cycle in ozone ground level concentration. Therefore, it is likely this station can be affected by a nearby unknown anthropogenic emission source, despite being listed as EMEP site. G2-Vilanova is considered “rural-background” according to the objective classification despite that site is affected by emissions from a nearby wood board factory. Other two stations that could be discussed are B1-A Magdalena and C9-Mourence, characterized as “rural-regional” following the objective classification, despite being suburban stations affected by traffic emissions, as evidenced by their daily ozone cycles. Therefore, even though the disadvantages of application of the subjective classification, in this work, it was preferred to the objective classification, because of the accurate knowledge of the sites environment and the limited influence on the surrounding sources in this region. For the complementary air quality sites applied in this work, all of them are EMEP sites; so they are classified as background rural sites. 

Because the complex terrain in the area under study can affect the ozone dynamic, as it was studied in other regions [14], a level 2 classification dependant on the topography (Table 5) was also done. Finally, following the Decission 97/101/CE [47], a classification by influence, based on the main emission sources around the sites, was done. Results of the three classifications (level 1, level 2, and influence) are summarized in Table 6. Again, for other sites different than the reference sites, classifications provided by the network managers were applied.

## 4. Results

For the analysis of ozone trends in this region, first possible episodic relationships between the different reference sites are obtained. After that, general trends (annual, monthly, and daily) are derived from the sites measurements. 

### 4.1. Sites Relationships

The geographical location and characteristics of the five sites considered to identify ozone episodes in this region can drive to establish relationships between the ozone levels observed at all of them, which can affect the study of ozone trends. This dependence between sites was estimated by the Pearson correlation coefficient (*r*) of the hourly ozone measurements along ozone episodes from 2002 to 2007 [52]. Results are shown in Table 7.

The highest correlation (Table 7) is obtained between B1 and C9 site measurements, located in suburban environments, with similar altitude and traffic emissions influence. Then, their measurements will be less useful for the study of rural ozone trends. 

Lower but significant correlation (Table 7) is obtained between B2 and F2 site measurements, as rural sites with similar surroundings; however, their specific locations are different, with F2 on a valley side, and B2 at the top of a hill. Even though their measurements follow similar patterns, local phenomena and different sources influence could affect their ozone levels. Therefore, their measurements will be analyzed separately. 

F2 and G2 site measurements show lower but significant correlation (Table 7), as both can be considered as background stations. However, their different topography and some influence of traffic emissions at G2 site produce those differences. 

Other sites show poor correlations along all the episodes. However, in some of them B1, B2, and C9 sites showed high correlations, which can indicate a general rise of ozone levels in the region, not related to local effects site by site. This feature could be considered for episodic analysis in this region. 

### 4.2. Seasonal and Interannual Trends of Tropospheric Ozone

For this analysis, ozone measurements in the period 2002–2006 from the five reference sites and the three EMEP at the northwest of the Iberian Peninsula were considered. 

**Figure 5:**
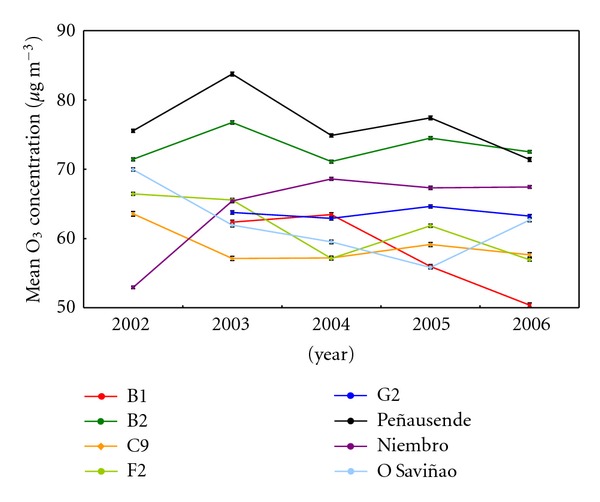
Series of annual mean 24-h ozone concentrations throughout the 5-year-study period (2002–2006) for each site.

For interannual trend, annual averages, 50 and 80 percentiles, were considered. Results are shown in Figure 5. Mean value along the 5 years moves between 50 and 73 *μ*g/m^3^ for the five reference sites, which are lower to the Peñausende site mean value (77 *μ*g/m^3^). On the other hand, F2, G2, Niembro, and O Saviñao sites show mean values around 63 *μ*g/m^3^, which are higher than B1 and C9 suburban sites mean values (58 *μ*g/m^3^), as expected. 

**Figure 6:**
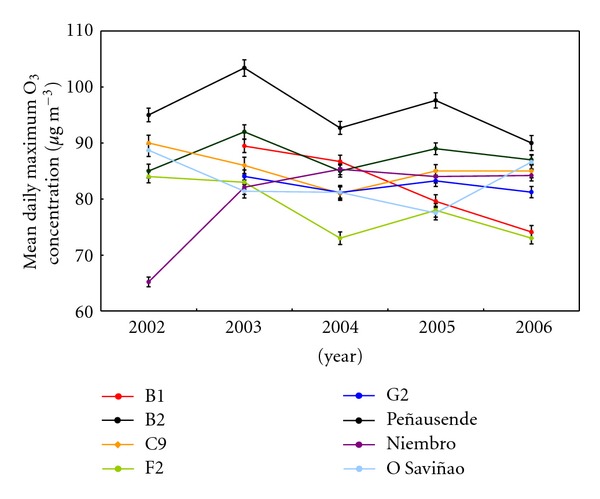
Series of annual mean of daily maximum ozone concentrations throughout the 5-year-study period (2002–2006) for each site.

Annual means of the maximum hourly data (Figure 6) show a range of 82–88 *μ*g/m^3^ at the five reference sites; as for the mean value, this range is lower to the mean of maximum at Peñausende (96 *μ*g/m^3^). 

For the reference sites, both mean and maximum ozone trends are similar, keeping small variations at rural stations; except on the year 2003, due to the effects of the forest fires in the Iberian Peninsula and the summer heat wave over Western Europe [53, 54]. Previous annual trends in this region [41, 55] show similar results. 

About EMEP sites, Peñausende shows the highest levels, due to its altitude (985 m) that reduces the ozone destruction by deposition and NO surface reaction, and increases its production because of the higher solar radiation [26, 56–58]. In fact, relatively higher means at B2 site should be related to its higher altitude, respect to the other reference sites, and the lower deposition over coastal areas. This effect is also observed at Niembro site, with a high annual mean; however, Niembro shows a low maximum daily annual mean, because of its low NO*_x_* level. O Saviñao shows an opposite behavior, because of the higher deposition in this inland location. 

G2 site shows a behavior similar to Niembro, because of its proximity to the coast; in the middle, between Niembro and O Saviñao, we can find B2 and F2 sites. Finally, B1 and C9, as inland suburban sites with traffic NO emissions and lower deposition close to the sea [59–61], show annual means lower than in rural sites, while maximum annual means are higher than F2 and G2 values; only B2 gets both higher annual and maximum annual means higher, because of its higher altitude. 

**Figure 7:**
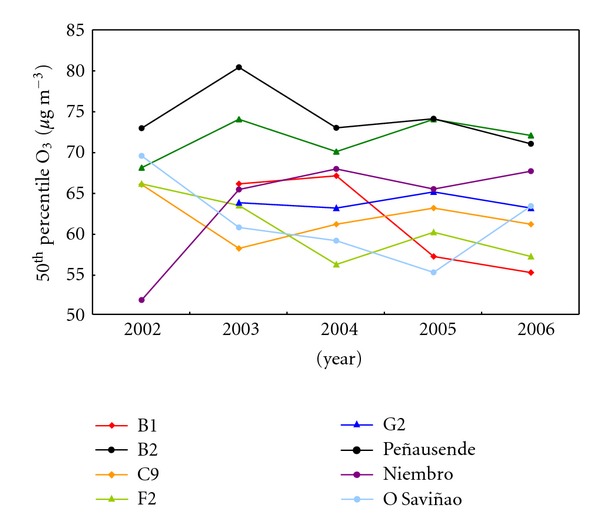
Internannual variability of tropospheric ozone concentration represented by the 50th percentile throughout the 5-year-study period (2002–2006) for each site.

Interannual trends were studied following a nonparametric test of Mann-Kendall [62], with 50th and 98th percentiles. The first percentile shows lower sensitivity to changes in emissions than the last one. 50th percentiles (Figure 7) show an increment at Niembro and a small reduction at Peñausende, considering the maximum achieved in 2003. More significant is the decrease at B1 site. 

**Figure 8:**
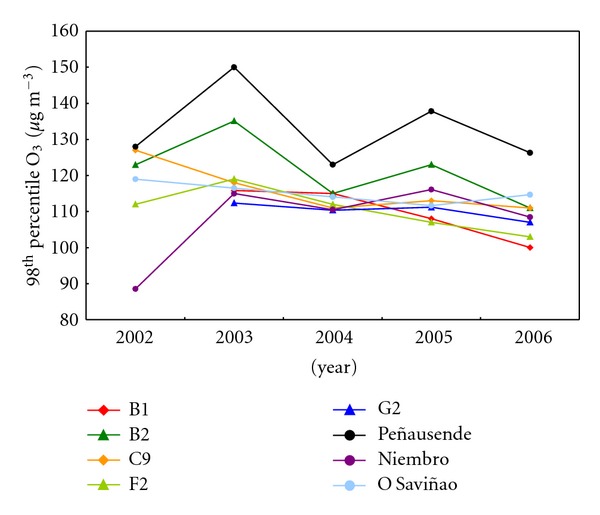
Internannual variability of tropospheric ozone concentration represented by the 98th percentile throughout the 5-year-study period (2002–2006) for each site.

Again, 98th percentiles (Figure 8) show a significant decrease at B1 site. These results are in agreement to the small variations of annual means but show a small trend to the ozone levels reduction. However, other studies covering global European trends from 1990 to 2004 [63, 64] show a 50th percentile stable or a bit rised (especially, during winter), and significant reductions in 98th percentile. Both European trends are related to the general NO*_x_* emissions reduction, that reduces both the ozone peaks [8, 9], and the available NO to destruct ozone during winter [65–67], increasing the background ozone, although this trend seems to be changed after the year 2000 [5]. 

These differences in the ozone trends between the northwest of the Iberian Peninsula and the rest of Europe can be explained because of the increment of NO*_x_* emissions at the Iberian Peninsula: from 1990 to 2006 NO*_x_*, emissions have increased 19% at Spain and 5% at Portugal [68, 69]. This can explain the stability of annual means and percentile 50 at this region, because of the levels stability. 

The slight decline in the 98th percentile between 2002 and 2006 is in agreement with the small but steady reduction of the peak ozone concentrations recorded in events analyzed between 2002 and 2007. Three quarters of these high-ozone events in Northern Galicia were associated with synoptic atmospheric conditions characterized by a blocking anticyclone to the north of the Iberian Peninsula and a high-altitude ridge of high pressure that extended across the Peninsula from Africa, following Saavedra et al. [52]. This synoptic situation causes a prevailing eastern-southeastern synoptic circulation, characterized by high temperatures, low humidity, and light wind speed. Keeping in mind that the distribution of pollutants is not only dependent on the spread of its emissions but is also affected by various weather/climatic drivers cannot be excluded that the decrease in 50th and 98th percentiles is also affected by changes in the large-scale circulation patterns over the NW Iberian Peninsula [70, 71], in turn related to the real-physical local circulations and weather types. Changes in the frequency of synoptic patterns favorable for high-ozone events could contribute significantly to the alteration of ozone trends [72]. However, the time series analyzed in this work are not long enough to establish a relationship between changes in tropospheric ozone concentrations with alterations in global synoptic circulation patterns. 

The annual cycle of tropospheric ozone at Northern Hemisphere has been widely studied, with seasonal variations and patterns related to the latitude and altitude [60, 73, 74]. At Europe, maximum levels are usually achieved in summer at Mediterranean regions and Central Europe because of photochemical production [75]. Otherwise, spring peaks are more usual at the Atlantic coast and the West of Europe [60, 76], as a general trend in the Northern Hemisphere [76–80]. 

**Figure 9:**
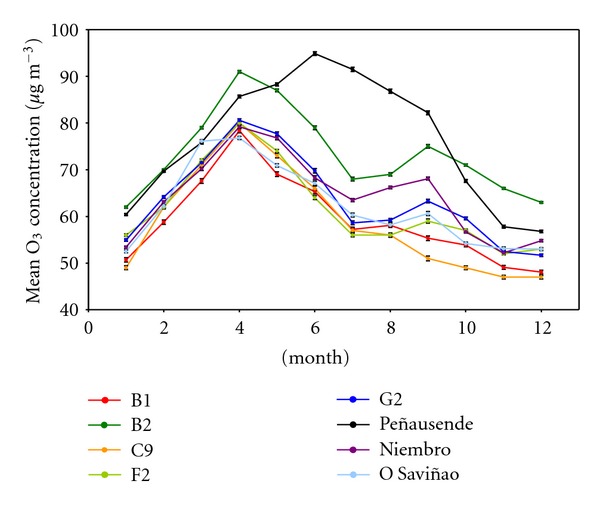
Series of monthly mean 24-h ozone concentrations throughout the 5-year-study period (2002–2006) for each site.

**Figure 10:**
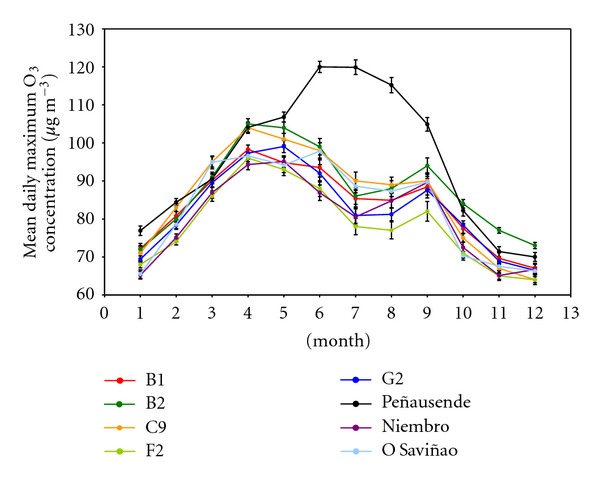
Series of monthly mean of daily maximum ozone concentrations throughout the 5-year-study period (2002–2006) for each site.

In the region under study, the five reference sites, Niembro and O Saviñao EMEP sites, show the typical seasonal cycle of boundary layer ozone for monthly daily mean (Figure 9) and monthly maximum mean (Figure 10) in low polluted regions of Northern Hemisphere. Both figures show a maximum on April, a relative minimum on July, and a minimum during winter. It is the same behavior of the ozone levels at Mace Head site and other Northern and Western European sites [5, 60, 65, 76, 81, 82]. In fact, there are evidences of the reduction of ozone levels at Mace Head after the year 2000 [5], which is also observed in the sites under study. At the same time, a significant contribution of transoceanic ozone was observed at Mace Head, as in the West Iberia [83], with some additional contribution of the stratospheric jet. The influence of ozone transported from the free troposphere toward the surface due to troposheric folding by high frequencies of troughs and cut-off lows in the Atlantic Iberian Coast must be taken into account, due to the high frecuency of these closed lows in the vicinity of the area under study, specially in summer [84, 85], and its clear impact over the surface ozone levels [55, 86–89]. This similar behavior of the sites under study can be explained because all these West Coast European sites are low sensitive to the reduction of NO*_x_* emissions at Central Europe [76]. 

A different behavior is observed at Peñausende site (987 asl-m) showing a typical pattern of elevated inland sites, with maximum ozone in June, persistence during summer, and slow decrease to the winter minimum [60]. These elevated sites are affected by the ozone at the free troposphere or, at least, accumulated in the upper boundary layer. 

Daily maximum monthly cycle (Figure 9) shows a similar behavior to the daily mean values cycle, especially at rural sites. Also B1 and C9 sites, with significant anthropogenic influence, show the highest values in winter, as the ozone destruction by the NO emissions does not affect the daily maximum. 

### 4.3. Daily Cycle Variability of Tropospheric Ozone

**Figure 11:**
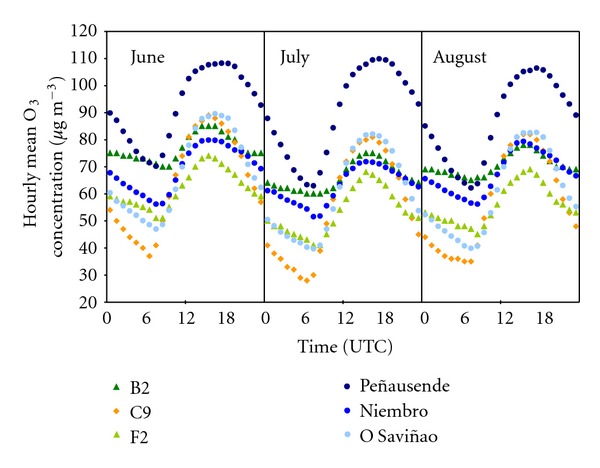
Series of hourly mean ozone concentrations in summertime (June, July, and August) throughout the 5-year-study period (2002–2006) for each site.

Summer is the best season for studying the daily cycle of tropospheric ozone [90, 91], as the period with the most favorable meteorological conditions for ozone peaks: high solar radiation and thermal inversions. From the analysis of the summertime daily cycles in the selected sites (Figure 11), three different patterns were identified.

- *Oscillated Cycle*. A daily cycle with strong oscillations, starting with a minimum at sunrise, with a quick increment along the morning due to the surface mixing (breaking the nocturnal thermal inversion) and the photochemical production. This trend is slower after noon, as the mixing layer growth, reaching the ozone peak around 17 UTC; after then, ozone level quickly falls down. This cycle is typical at Peñausende and O Saviñao sites, two inland rural sites with significant altitudes.
- *Suburban Cycle*. A similar but less oscillating cycle, starting again with a minimum at sunrise, with a quick increment until 15 UTC, and a softer decrease along the afternoon. Although this cycle could be similar to the previous one, it is typical at suburban sites (B1, C9), and related to the local NO emissions that destruct the ozone at night, and to a faster growth of the nocturnal stable layer, which is more favorable to the ozone elimination.
- *Nonoscillated Cycle*. A soft daily cycle, with a minimum around sunrise, but similar to the previous nocturnal levels. The ozone peak can be achieved between 14 and 16 UTC, falling down quickly until 22 UTC, when the nocturnal level keeps quite constant. B2 and Niembro sites clearly follow this pattern; F2 and G2 sites show a bit strong oscillations, as an intermediate behavior between soft and suburban cycle.

**Figure 12:**
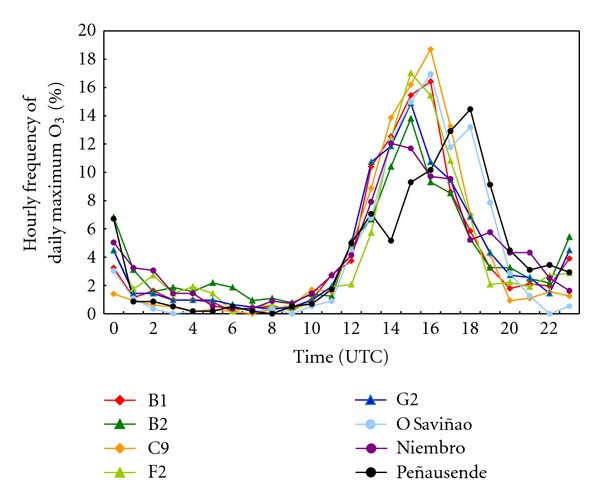
Frequency distribution of daily maximum hourly ozone in summertime (June–August) during the period 2002–2006.

In this region, coastal sites show a soft daily cycle, due to the winds around them which promote unstability [61, 92], mixing the surface ozone. In addition, if the NO*_x_* nocturnal levels at these sites are low, the ozone destruction by NO is slow [59], increasing the ozone levels. This unstability nocturnal effect is especially significant at B2 site, because of its altitude favorable to strong nocturnal winds to the valley. In fact, in the hourly frequency of ozone peaks (Figure 12), small nocturnal ozone peaks can be observed, due to the mixing between surface and aloft layers with ozone [93]. 

About the absolute daytime ozone peaks, B2, G2, and Niembro sites show peaks around 14-15 UTC, which is earlier to the inland peaks at Peñausende (17-18 UTC). As daytime peaks mainly depend on photochemical production, and solar irradiance is similar in these sites, only the higher maximum summertime temperatures at Peñausende [94, 95] can explain this difference in ozone production. 

At night, relative differences between coastal and inland sites are even more significant: B2, F2 and Niembro sites (Figure 12) show significant frequencies of nocturnal peaks (20–25%), whereas C9 and O Saviñao inland sites show less than 8%. These nocturnal peaks appear in the interval 21-00 UTC, when mixing layer depth is decreasing and nocturnal stable layer is growing [96]. At B2 and F2 sites, this behavior can be explained by the downhill nocturnal winds, which carry ozone from upper layers [58, 93]. The pure coastal Niembro site is affected by the inland nocturnal breezes, which carry ozone from the top of hills close to this site.

Some nocturnal peaks can be also observed at Peñausende inland site, due to its high altitude and surrounding mountains, which develop the same phenomena as at B2 and F2 sites.

All these nocturnal differences are enhanced by the low destruction of ozone at rural sites, as B2, F2, Niembro, and Peñausende [97]. 

## 5. Conclusions

Long-term trends of tropospheric ozone studied in the past over diverse regions of Western Europe show a different behavior of Atlantic sites. Particularly, different trends were identified at several coastal regions of the Iberian Peninsula (Mediterranean, Cantabric, Southwestern). In this work, trends at northwestern part of this Peninsula from 2002 to 2007 were analyzed and compared to previous studies over boundary regions. 

About annual trends, both mean and maximum values are quite stable, except in 2003, because of the influence of forest fire emissions over the Iberian Peninsula, and the summertime heat wave over Western Europe. 98th percentile shows a small ozone reduction, in agreement to the stronger global reduction over Europe. Smaller reduction over the region under study can be explained by the increment of NO*_x_* emissions from Spain and Portugal during that period. Seasonal trend in this region is in agreement to trends in low polluted regions of Northern Hemisphere, because of the significant contribution of transoceanic pollution already identified in other reference sites (i.e., Mace Head). In fact, an inland site close to this region shows a different behavior. 

About daily ozone cycle, from the analysis of summertime series, three different patterns were identified: oscillated, nonoscillated, and suburban, as intermediate between both. In this region, oscillated cycle was typical in inland elevated sites; nonoscillated cycle was observed in coastal sites (up to 30 km far from the sea), with more oscillation as the site was far from the coast; and suburban cycle was observed in coastal sites with NO local emissions; maximum daily ozone during summertime confirms this trend. Apart from it, the observation of relative peaks of nocturnal ozone at elevated sites is a specific phenomena that is usually associated to the injection of ozone from upper tropospheric layers; so it is not related to the photochemical activity in this region. 

Considering just the available rural stations, the ozone trends observed in this study could be extrapolated to other rural areas of NW Iberian Peninsula, highlighting the Galician Coast and the northern coast of Portugal, because this geographical area has remarkably homogeneous topographical and climatological characteristics. In addition, the Galician-Portuguese Atlantic Coast is affected by the same phenomena of long-range pollutant transport, due to synoptic circulation patterns characteristic of this area. However, these ozone trends are linked to the interannual meteorological variability, which suffer significant changes during the period under study; in addition, changes in the photochemical precursor emissions over this region and its surroundings also affect those trends. The analysis of future ozone trends in this region should be very useful to confirm the influence of these factors. 

Because of the small number of representative sites and the short time series available for this analysis, the systematic application of regional air quality models [98] over this region would help to improve and extend the understanding of regional surface ozone trends.

## Figures and Tables

**Table 1:** Summary of exceedances of the ozone thresholds stablished by the Ambient Air Quality and Cleaner Air for Europe Directive [39] in five stations at the Northwestern Iberian Peninsula during the period 2003–2008. Source [40].

Year	Daily maximum of 8-h mean > 120 *μ*g/m^3^		Hourly mean > 180 *μ*g/m^3^		
	Number of days	3 years average	No. of days	No. of hours	No. of stations
2003	No data	—	5	11	4
2004	No data	—	3	8	3
2005	No data	—	0	0	0
2006	No data	—	0	0	0
2007	26	—	0	0	0
2008	6	—	0	0	0

**Table 2:** Air quality stations whose data have been used for comparative purposes in this study. The coordinates are Universal Transverse Mercator (UTM) and grid zone 29T, except Peñausende and Niembro stations, in grid zone 30T.

Station	UTMx (km)	UTMy (km)	Distance to the sea (km)	Altitude (m)	Monitoring network
ES16-O Saviñao	606.1	4721.2	88.1	506	EMEP
ES08-Niembro	350.3	4811.7	0.0	134	EMEP
ES13-Peñausende	259.9	4574.3	238.0	985	EMEP
B1-A Magdalena	593.3	4811.4	28.0	363	Regional
B2-Louseiras	601.8	4821.1	18.0	540	Regional
C9-Mourence	606.0	4796.4	41.0	465	Regional
F2-Fraga Redonda	581.9	4806.3	16.0	480	Regional
G2-Vilanova	578.5	4822.9	11.0	290	Regional

**Table 3:** Percentage of valid hourly O_3_ measurements per year and station. Data of the the year 2007 are refered to its first eight months.

Station	Data capture (%)					
	2002	2003	2004	2005	2006	2007^(∗)^
B1-A Magdalena	47.9	99.1	95.0	92.7	98.1	97.3
B2-Louseiras	94.7	96.5	95.1	96.7	98.5	99.5
C9-Mourence	96.4	98.8	99.4	98.3	98.8	99.0
F2-Fraga Redonda	95.7	98.7	94.6	96.1	98.7	96.6
G2-Vilanova	50.2	99.4	98.3	99.2	98.7	97.2
O Saviñao	97.7	95.9	97.6	97.9	96.6	96.3
Niembro	97.5	92.2	97.5	97.6	98.5	99.0
Peñausende	93.3	92.4	97.7	96.7	97.1	98.1

*Year 2007 only includes the first eight months.

**Table 4:** Classification of air quality monitoring stations following the *subjective* [51] and *objective* level 1 classification [46].

	Subjective classification [51]		Objective classification [46]		
Station	Type	Influence	Type	[O_3_]/[O*_x_*] ratio	
				Summer (max.)	Winter (min.)
B1-AMagdalena	Suburban	Traffic	Rural-regional	0.95	0.67
B2-Louseiras	Rural	Background	Background rural	0.97	0.92
C9-Mourence	Suburban	Traffic	Rural-regional	0.95	0.76
F2-Fraga Redonda	Rural	Background	Background rural	0.97	0.87
G2-Vilanova	Rural	Industrial	Background rural	0.95	0.81
O Saviñao (EMEP)	Rural	Background	Rural-regional	0.96	0.75
Niembro (EMEP)	Rural	Background	Background rural	0.97	0.80
Peñausende (EMEP)	Rural	Background	Background rural	0.98	0.80

**Table 5:** Classification of air quality monitoring stations, namely, Level 2, according to their topographic environment.

Level 2	Orographic location
EI	Elevated inland
IC	Intermediate elevated mountain slope between coast and inland
CL	Coastal low altitude
IM	Intermediate elevated mountain slope at inland

**Table 6:** Final classification of air quality monitoring stations; measurements from 2002 to 2007 were used for statistical ozone analysis.

Station	Level 1	Level 2	Influence
B1-AMagdalena	Suburban	IM	Traffic
B2-Louseiras	Rural	IC	Background
C9-Mourence	Suburban	IM	Traffic
F2-Fraga Redonda	Rural	IC	Background
G2-Vilanova	Rural	CL	Industrial
O Saviñao	Rural	IM	Background
Niembro	Rural	CL	Background
Peñausende	Rural	EI	Background

**Table 7:** Correlations of ozone levels between the five air monitoring stations during ozone episodes, using the Pearson correlation coefficient *r*. Periods containing less than 80% of the total data were withdrawn from the analysis.

Pair of stations	Pearson coefficient (*r*)			
	Mean	Standard deviation	Min.	Max.
B1-B2	0.48	0.18	0.18	0.85
B1-C9	0.87	0.05	0.68	0.93
B1-F2	0.63	0.17	0.19	0.90
B1-G2	0.68	0.12	0.33	0.87
B2-C9	0.49	0.16	0.09	0.77
B2-F2	0.78	0.15	0.17	0.92
B2-G2	0.68	0.16	0.29	0.89
C9-F2	0.61	0.14	0.20	0.82
C9-G2	0.63	0.11	0.32	0.81
F2-G2	0.72	0.13	0.44	0.92
